# Inter-rater Reliability and Accuracy of NBI Score for Activity Evaluation in Ulcerative Colitis

**DOI:** 10.2174/0115748871367645250623062144

**Published:** 2025-07-03

**Authors:** Gianpiero Stefanelli, Marco Valvano, Annalisa Capannolo, Stefano Necozione, Angelo Viscido, Giovanni Latella, Michele Marchese

**Affiliations:** 1 Gastroenterology Unit, Galliera Hospital, Via Mura delle Cappuccine 14, 16128, Genova, Italy;; 2 Department of Life, Health and Environmental Sciences, Gastroenterology Unit, University of L’Aquila, Piazzale Salvatore Tommasi 1, 67100, L’Aquila, Italy;; 3 Diagnostic and Surgical Endoscopy Unit, San Salvatore Academic Hospital, 67100, L’Aquila, Italy;; 4 Epidemiology Unit, Department of Life, Health and Environmental Sciences, University of L’Aquila, 67100, L’Aquila, Italy

**Keywords:** Inflammatory bowel disease (IBD), narrow banding imaging (NBI), ulcerative colitis, virtual chromoendoscopy, endoscopy, Mayo score, Mayo Endoscopic Score (MES)

## Abstract

**Introduction:**

Colonoscopy is a critical tool for the management of Ulcerative Colitis (UC). In this study, we aim to explore the accuracy of Virtual Chromoendoscopy (VCE) using Narrow Banding Imaging (NBI), and magnification using Near Focus (NF) for a better definition of endoscopic inflammation grade in UC patients compared to standard white light (WL) endoscopy alone.

**Methods:**

This is a non-randomized prospective study including all the patients who underwent a colonoscopy (for any reason) between April and September 2023 (with protocol number n. 60/2019.20). During the endoscopic evaluation, at least one image with white light - evaluated using the Mayo Endoscopic Score (MES), one image with NBI, one image with NBI plus NF, and a biopsy were obtained in each colonic tract (cecum, ascending colon, transverse colon, descending colon, sigmoid colon, and rectum). All the stored images were evaluated by three endoscopists separately and compared with the results of the histological evaluation.

**Results:**

A total of 31 UC patients were included. The inter-rater reliability concerning the different scores used (MES, NBI score, and NBI plus NF score), which was evaluated with Cohen's kappa coefficient, was good for the MES, good to excellent for the NBI score, and good for the NBI plus NF score. The concordance between histological evaluation (using the Nancy Index) and the MES was unsatisfactory for all the endoscopists, while the concordance between the NBI evaluation score and the Nancy Index was good to excellent.

**Discussion:**

The reported results demonstrated both the inter-rater reliability and diagnostic accuracy of NBI in assessing endoscopic disease activity. The high inter-operator agreement, ranging from good to excellent, further supports the feasibility and consistency of NBI as a practical tool.

**Conclusion:**

The results in the present study suggest that the new endoscopic technologies could be useful to better define disease activity in Inflammatory Bowel Disease (IBD) patients driving better therapeutic strategy.

## 1 INTRODUCTION

Ulcerative colitis (UC) is an idiopathic inflammatory disease that affects the colon. Colonoscopy is a critical tool both for the diagnosis of UC and for the surveillance of associated dysplasia/colorectal carcinoma (CRC) [1]. Moreover, colonoscopy plays a crucial role in assessing the degree of mucosal inflammation, allowing for therapy adjustments aimed at achieving the key objective of mucosal healing within the “treat-to-target” strategy [2]. Currently, assessing the therapeutic efficacy based solely on clinic and laboratory parameters is considered suboptimal and inadequate. Conversely, the optimal therapeutic goal should encompass both endoscopic and histological remission [3, 4]. Microscopic inflammation is related to the UC prognosis (*e.g*. risk of relapse, hospitalization, dysplasia, and CRC) [5, 6]. The risk of 12 months of relapse is 52% in patients with acute inflammatory infiltrates, compared to 25% in those without the disease [2]. Thus, the endoscopic revaluation about 6 months later (from 3 to 9 months) after initiating or modifying therapy is recommended [2]. Moreover, recent studies have suggested that achieving a Mayo Endoscopic Subscore (MES) of 0 may provide additional benefits in reducing clinical relapse compared to MES 1 [7, 8]. However, the most widely accepted definition of endoscopic remission used in clinical trials includes both MES 0 and 1 [9].

Routine colonoscopy is performed with white light (WL), but emerging endoscopic technologies such as high definition (HD), virtual chromoendoscopy (VCE), and magnification provide the opportunity to study the mucosa more accurately. In the surveillance of dysplasia and CRC associated with UC, guidelines recommended the use of VCE and HD endoscopy with target biopsies, rather than WL endoscopy with random biopsies [1, 10]. Conversely, the potential role of these emerging endoscopic technologies in more precisely assessing UC inflammation compared to WL alone, as well as their feasibility in routine clinical practice, remains a topic of ongoing debate.

Some studies have emphasized the role of VCE and HD to grade UC activity accurately [3, 4]. In the actual scenario of a “treat-to-target” strategy that requires verifying the achievement of endoscopic remission, these tools could improve daily practice. Moreover, HD, VCE, and magnification could help discriminate the different degrees of MES precisely, and they could have the potential role in predicting the histological activity grade.

In conclusion, VCE and HD have a consolidated role in the detection and characterization of colonic [11] and gastric lesions [12], and dysplasia and CRC associated with UC [13, 14]. However, their role in the evaluation of UC inflammation is uncertain [4].

This study aims to evaluate the role of VCE using Narrow Banding Imaging (NBI) and magnification with Near Focus (NF) in improving the assessment of endoscopic inflammation severity in UC patients compared to WL alone.

We analyzed both the inter-rater reliability and the accuracy of inflammation endoscopic scores determined with the use of NBI and NF.

## 2 METHODS

This is a non-randomized prospective study.

All the patients who underwent colonoscopy (for any reason) between April and September 2023 at the Diagnostic and Surgical Endoscopy Unit of San Salvatore Academic Hospital in L’Aquila (Italy) were considered eligible. Among them, all IBD patients, including those with a pre-existing diagnosis as well as those with a newly diagnosed condition, were deemed eligible for inclusion.

All clinical investigations were conducted according to the principles stated in the Declaration of Helsinki. The results were reported according to the Strengthening the Reporting of Observational Studies in Epidemiology (STROBE) Statement guidelines [15]. Ethics approval was issued by the Internal Review Board of the University of L’Aquila (protocol number n. 60/2019.20). Informed consent was obtained from all subjects for participation in the current study.

### 2.1 Inclusion Criteria

The inclusion criteria were as follows:

- Patients who underwent colonoscopy for any indication between April and September 2023,

- Definitive diagnosis of UC based on standard clinical practice and histological definition according to current guidelines [16],

- Age ≥ 18 years old,

- Adequate bowel preparation (Boston scale ≥ 6 and ≥ 2 in any of the colonic tract) at the colonoscopy [17],

- Complete examination (caecal intubation) [18].

### 2.2 Exclusion Criteria

The exclusion criteria were as follows:

- Diagnosis of IBD different from UC,

- Lack of use of a colonoscope equipped with VCE (NBI) and magnification (NF) for any reason.

### 2.3 Outcome of Interest

Our primary outcome was to evaluate the concordance between the NBI and NBI plus NF scores and the histological activity. Moreover, we assessed the accuracy of all evaluated scores as well as the inter-rater reliability of the different scoring systems and endoscopist evaluations.

### 2.4 Medical Records

For all the included patients, the following data were collected:

- Demographic characteristics (sex, age);

- Disease characteristics (date of diagnosis, Montreal classification [19], pharmacological therapies, disease activity expressed as Partial Mayo Score [20]);

### 2.5 Endoscopic Procedure

#### 2.5.1 High-definition, Virtual Chromoendoscopy, Magnification

For all colonoscopies, the Olympus CF-HQ190I/L colonoscope, equipped with HD, magnification function (NF), and NBI was used.

The resolution of an optical system refers to its ability to distinguish between two closely spaced points. Higher resolution corresponds to a greater number of pixels, resulting in enhanced image quality. High-definition (HD) endoscopy provides a resolution exceeding 1,000,000 pixels, significantly improving visualization and diagnostic accuracy [21].

The magnification function is a system using a variable dual-focus lens system that can closely (from 2 to 6 mm) inspect a target area of the mucosa, maintaining focus. Thus, it is defined dual or NF system and it allows magnification up to 70 times [21, 22].

VCE is a real-time and on-demand technology that has replaced vital chromoendoscopy almost completely. NBI by Olympus is a type of VCE that recurs to optical filtering of WL selecting two specific wavelength ranges (or bands): 415 nm - blue spectrum of light; and 540 nm - green spectrum of light. These two wavelength bands correspond to the main peaks on the absorption spectrum of hemoglobin: the first (415 nm) is less penetrating and allows us to see mucosal capillaries that appear in brown; the second (540 nm) penetrates deeper and allows us to see deeper mucosal and submucosal vessels that appear in cyan [23]. Thus, NBI - alone or in association with HD and magnification - improves the study of vascular and mucosal patterns [10, 21, 23, 24].

Prior to patient enrollment, all endoscopists involved in the study underwent a one-month training period, during which they practiced image interpretation by performing colonoscopies together.

#### 2.5.2 Images and Biopsies Collection

During the endoscopic evaluation, at least one image with WL, one image with NBI, and one image with NBI plus NF were obtained in each colonic tract (cecum, ascending colon, transverse colon, descending colon, sigmoid colon, and rectum). A biopsy was obtained after capturing the three-image sequence. Thus, for each evaluated colonic tract, at least three images and one corresponding biopsy were collected for histological evaluation. Notably, each image and biopsy were acquired at the same location. Each collected biopsy wasretained in a distinct cuvette linked to the corresponding three-image sequence with a unique identification number. At the end of the endoscopy, all images were systematically stored for further analysis.

### 2.6 Images Evaluation

At the end of the image collection phase, all the stored images were evaluated by three endoscopists separately:

- Endoscopist 1 was the senior (more than 20,000 procedures performed in the professional career);

- Endoscopist 2 had a medium-high experience (more than 10,000 procedures performed in the professional career);

- Endoscopist 3 was in advanced training (more than 1,000 procedures performed in the professional career).

The patients' identity, disease history, and the decision concerning the evaluation of the images made by other endoscopists were not known by the others.

Three different scores were used for the evaluation of the endoscopic activity. Images obtained with WL were evaluated with MES [25]. For each image obtained with NBI evaluation (both alone and with NF), two proposed scores were used: NBI score and NBI plus NF score, respectively, adapting two models already present in the literature [26, 27]. Four degrees of freedom were allowed for each score (0: remission; 1: mild activity; 2: moderate activity; 3: severe activity).

The characteristics of the different scores used for the evaluation of the images are reported in Table **1**. Sample images for each of the inflammation degrees are shown in Figs. (**1** and **2**).

### 2.7 Histologic Evaluation

The histological activity of the collected sample was evaluated according to the Nancy Index (NI) [28]. Four degrees of histological activity were allowed as shown in Table **2**.

### 2.8 Statistical Analysis

All the statistical analyses were performed using the statistical software SAS 9.4 (SAS Institute Inc).

Data were summarized using absolute and relative frequencies for categorical variables and median and range for numerical variables.

The agreement between different endoscopists was explored to establish inter-rater reliability. The concordance between endoscopic and histological inflammation scores was investigated to define the test accuracy. The inter-rater reliability concerning the different scores used (MES, NBI evaluation score, and NBI plus NF evaluation score) was evaluated with Cohen's kappa coefficient. Moreover, the concordance between histological evaluation (using the NI) and the endoscopic scores or clinical activity (evaluated with the Partial Mayo Score) was also assessed with Cohen's kappa coefficient.

The indices of the agreement were evaluated with the following landmarks:

• k < 0.01: no agreement;

• 0.01 < k < 0.20: poor agreement;

• 0.21 < k < 0.40: small agreement;

• 0.41 < k < 0.60: mild agreement;

• 0.61 < k < 0.80: good agreement;

• 0.81 < k < 1.00: excellent agreement [29].

## 3 RESULTS

During the enrollment period, a total of 474 colonoscopies were performed. Out of 474 patients, who underwent colonoscopy, 50 IBD patients were deemed eligible for inclusion (38 UC and 12 Crohn’s Disease, respectively). A total of 19 patients were excluded: 12 Crohn’s Disease patients and 7 UC patients because they did not fulfill the inclusion criteria (5 BBS < 6 at the colonoscopy, 1 undetermined Colitis, 1 exam performed with a colonoscope without NF technology due to a colon sub-stenosis and the necessity to use an ultra-slim endoscopy).

Fig. (**3**) shows the enrolment flow chart and image acquisition.

### 3.1 Baseline Characteristics

**Fig. (3):**
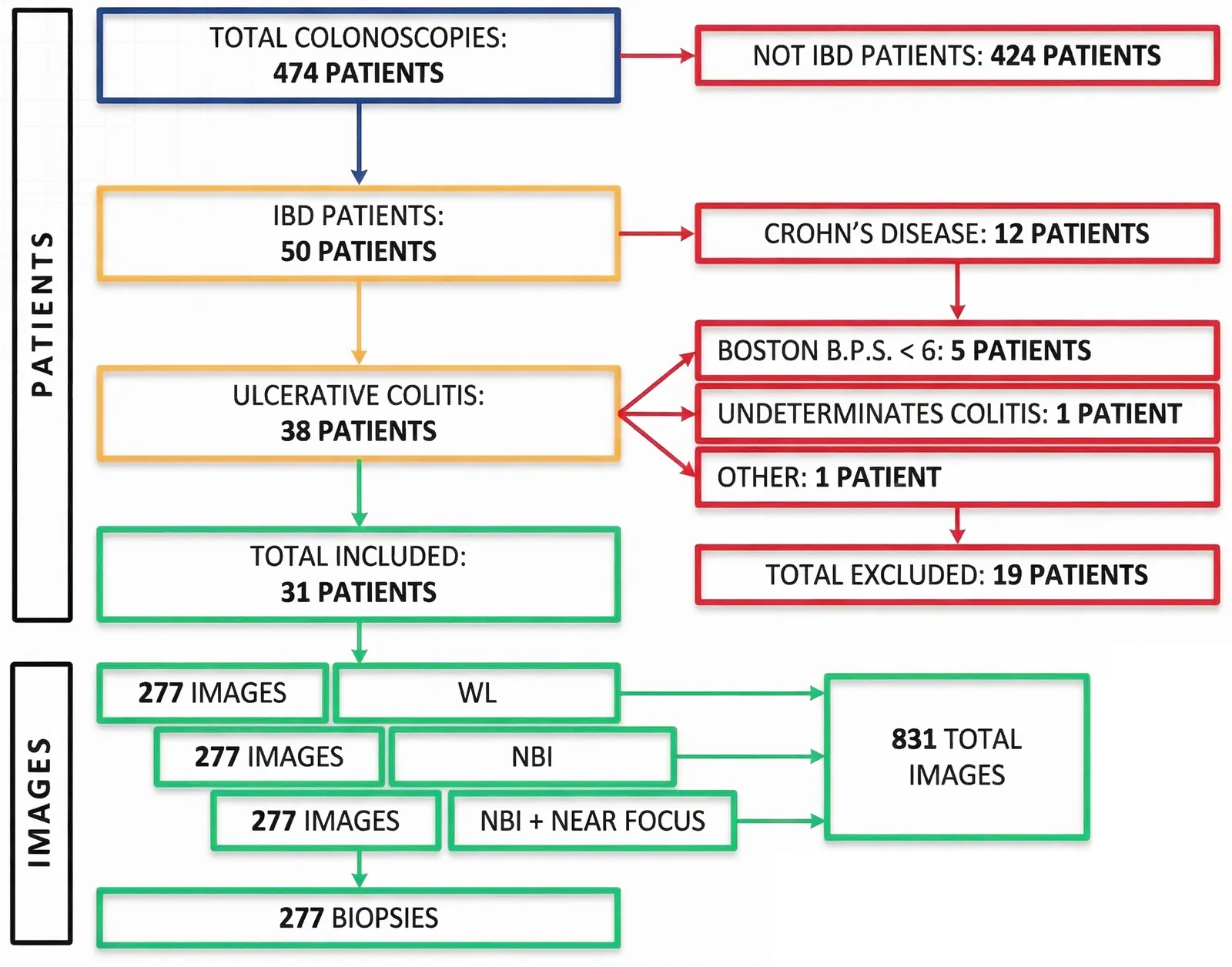
Enrolment flow chart and image acquisition.

Among the 31 patients included in the final analysis (17 male and 14 female - median age 48 years; range 21-80), a total of 831 images were captured and examined (Fig. **3**).

The distribution of UC cases was as follows: 20 extensive colitis, 9 left-side colitis, and 2 ulcerative proctitis.

Additionally, 4 of the 31 included patients were newly diagnosed with UC at the time of colonoscopy during enrollment (overall median disease duration: 9 years, range: 0-40). The baseline characteristics of the included patients and the prescribed therapies were reported in Table **3**.

### 3.2 Histological and Endoscopic Concordance

The concordance between histological evaluation (using the NI) and the MES was unsatisfactory for all the endoscopists (k= 0.40 - 95% CI, 0.33-0.46; k= 0.35 - 95% CI, 0.28-0.41; k= 0.34 - 95% CI, 0.28-0.41).

The concordance between the NBI evaluation score and the NI was good-excellent (for the senior endoscopist k= 0.78 - CI 95%, 0.72-0.83; for the medium-high experienced endoscopist k= 0.72 - CI 95%, 0.66-0.78, and for the trainer k= 0.81 - CI 95%, 0.76-0.86).

The NBI plus NF showed a small-mild concordance (for the senior endoscopist k= 0.42 - CI 95%, 0.35-0.49; for the medium-high experience endoscopist k=0.48 - CI 95%, 0.41-0.55; for the trainer k= 0.38 - CI 95%, 0.31-0.45) (Table **4**).

### 3.3 Inter-rater Reliability

The inter-rater reliability concerning the different scores used (MES, NBI score, and NBI plus NF score) evaluated with Cohen's kappa coefficient was good for the MES, good to excellent for the NBI score, and good for the NBI plus NF score (Table **5**).

In particular, the senior endoscopist showed an inter-rater reliability of 0.79 (95% CI, 0.73-0.85), 0.84 (95% CI, 0.79-0.89), and 0.77 (95% CI, 0.71-0.84) for the MES, NBI, and NBI plus NF score, respectively.

Moreover, the inter-rater reliability was excellent between the senior and the trainer endoscopist for the NBI and NBI plus NF scores (k= 0.91 - 95% CI, 0.87-0.95 and k= 0.87 - 95% CI, 0.82-0.92, respectively).

### 3.4 Histological and Clinical Concordance

The concordance between histological evaluation (using the NI) and clinical activity (using the partial Mayo Score) was poor (k= 0.08).

## 4 DISCUSSION

In the current“treat-to-target” approach [2], advanced endoscopic technologies such as HD, VCE, and magnification represent valuable tools for the management of IBD. Over the years, therapeutic goals have evolved from focusing solely on clinical and laboratory improvement to incorporating endoscopic and histological remission as key objectives [3, 4]. Moreover, achieving MES 0 rather than MES 1 has been associated with a more favorable prognosis in UC patients [8]. HD, VCE, and magnification enhance the visualization of colonic mucosa, allowing for a more precise assessment of disease activity compared to conventional WL endoscopy. These technologies may improve the discrimination of different degrees of MES, refining disease stratification and management strategies. Our study demonstrated both the inter-rater reliability and accuracy of NBI in assessing endoscopic disease activity. The higher inter-operator-agreement (ranging from good to excellent) supports the ease of use of this endoscopic tool in routine clinical practice. Thus, it indicates the feasibility of directness in daily clinical practice. The accuracy is shown by the concordance (ranging from good to excellent) between the histological activity (measured by NI) and endoscopic activity defined by NBI. Conversely, the evaluation of mucosa activity by WL indicated MES showed less accuracy: in fact, our data showed a small concordance between MES and NI, according to literature data [30]. The weak correlation between MES, which is based solely on WL observation, and histology suggests that MES may be insufficient for accurately defining disease activity. Our findings indicate that NBI provides valuable insights into histological activity. In the future, we hope for the development of a validated, modified MES incorporating NBI features to enhance the assessment of UC disease activity.

Moreover, our results demonstrated that NBI is particularly able to better distinguish between the lower grades of mucosa inflammation (MES 0 *versus* 1). Comparing the evaluation by WL and NBI, the number of images categorized as MES 0 by WL decreased after the NBI evaluation: in fact, 158 images were categorized as MES 0 at WL, compared to 113 cases categorized as NBI 0 (for the endoscopist 1). Consequently, NBI evaluation increased the number of images classified as a mild grade of inflammation: from 79 cases of MES 1 to 101 cases of NBI 1 (for the endoscopist 1). The data for endoscopists 2 and 3 are analogous. Similarly, NBI evaluation increased the number of moderate and severe grades of inflammation, but less significantly. Thus, these data support the idea that NBI evaluation can acquire much more information as compared to WL evaluation. Conversely, WL evaluation alone could provide an understanding of the real grade of mucosa inflammation. Considering that MES 0 could be a more ambitious target compared to MES 1 [7, 8], these data are very significant [2].

A more exact definition of mucosa inflammation grade allows the therapy immediately at the end of the endoscopy, avoiding waiting for the histological report. While NBI has demonstrated superiority over WL endoscopy in defining the degree of inflammation, it is not intended to replace biopsies. Instead, NBI serves as a valuable adjunct, providing real-time insights and enabling targeted biopsies from the most visibly inflamed areas. This approach ultimately enhances the accuracy of inflammation grading, optimizing both diagnosis and treatment strategies.

Although our results demonstrated good inter-rater reliability of NBI plus NF in the evaluation of the endoscopic activity degree, the results about its accuracy were disappointing (the concordance between the histological activity and endoscopic activity defined by NBI plus NF ranged from low to moderate). This discrepancy is likely attributable to certain study limitations. First, to define the endoscopic grade of inflammation by NBI plus NF evaluation, we adapted a model based on a Pentax endoscope equipped with i-SCAN [26]. Conversely, in the case of NBI, we used a score based on a model present in the literature and based on the Olympus endoscope [27]. Moreover, the usage habit of the VCE with NBI is higher than with magnification with NF. Consequently, the confidence in the use and interpretation of NBI technology is higher than in NF. The degree of confidence for the NBI evaluation is probably facilitated by the routine and consolidated practice of studying the vascular pattern to define MES during colonoscopy with WL. Thus, in the future, it would be desirable to improve the level of confidence with the use of NF technology, which would allow for improving the performance of the visualization combining NBI and NF.

Certainly, a further limitation of our study is the lack of data on colonoscopy duration; thus, we cannot conclude that using VCE is not significantly time-consuming. Although we did not analyze it, we can reasonably assess that VCE does not significantly lengthen the exam time, especially for the operator with a certain degree of experience in such display settings: in fact, it is immediate on-demand technology (pressing the relative activation key on the endoscope handle).

Interestingly, the poor concordance between histological evaluation (NI) and clinical activity (partial Mayo Score) confirms that the clinical laboratory monitoring alone is unacceptable and insufficient; thus, endoscopy plays a fundamental role in monitoring disease activity [3, 4].

Nonetheless, the excellent inter-rater reliability between the senior and trainee endoscopists indicates that the learning curve for VCE with NBI and magnification with NF is not particularly challenging. This finding supports its feasibility and potential for routine clinical practice.

## CONCLUSION

The findings of this study suggest that new endoscopic technologies, particularly the VCE visualization modality, may enhance the assessment of disease activity in IBD patients, ultimately guiding more effective therapeutic strategies. This is especially relevant in a treat-to-target approach and within the current landscape of increasingly ambitious therapeutic goals.

Further data, ideally from multicenter studies, are needed to confirm the accuracy and feasibility of this endoscopic tool.

## STUDY LIMITATIONS

Undoubtedly, the restricted sample size and non-randomized design represent significant limitations in generalizing the study results. Additionally, a multicenter study would enable the comparison of image interpretations by multiple endoscopists, thereby reducing interpretation bias and enhancing reproducibility. Such an approach is essential for obtaining robust evidence on this issue.

## AUTHORS’ CONTRIBUTIONS

The authors confirm their contribution to the paper as follows: analysis and interpretation of results: SN; draft manuscript: AC; Conceptualization: GS, MM; Methodology: MV; Validation: AV, GL; All authors reviewed the results and approved the final version of the manuscript.

## Figures and Tables

**Fig. (1):**
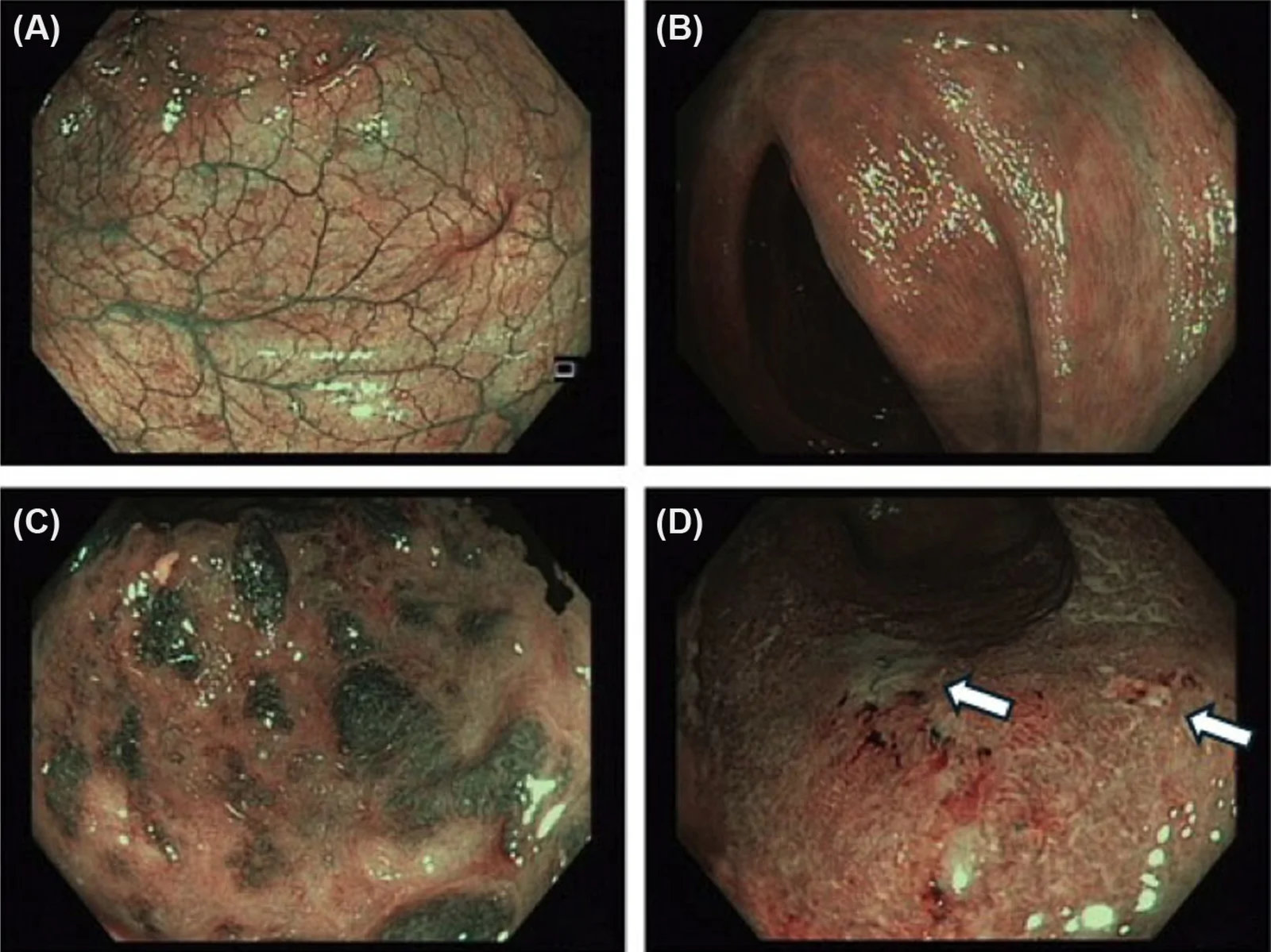
Images for each of the inflammation degrees with NBI evaluation. **A**: grade 0; **B**: grade - vascular pattern partially represented 1; **C**: grade 2 - not visible vascular pattern; **D**: grade 3 - vascular pattern not visible and ulcerations (dots).

**Fig. (2):**
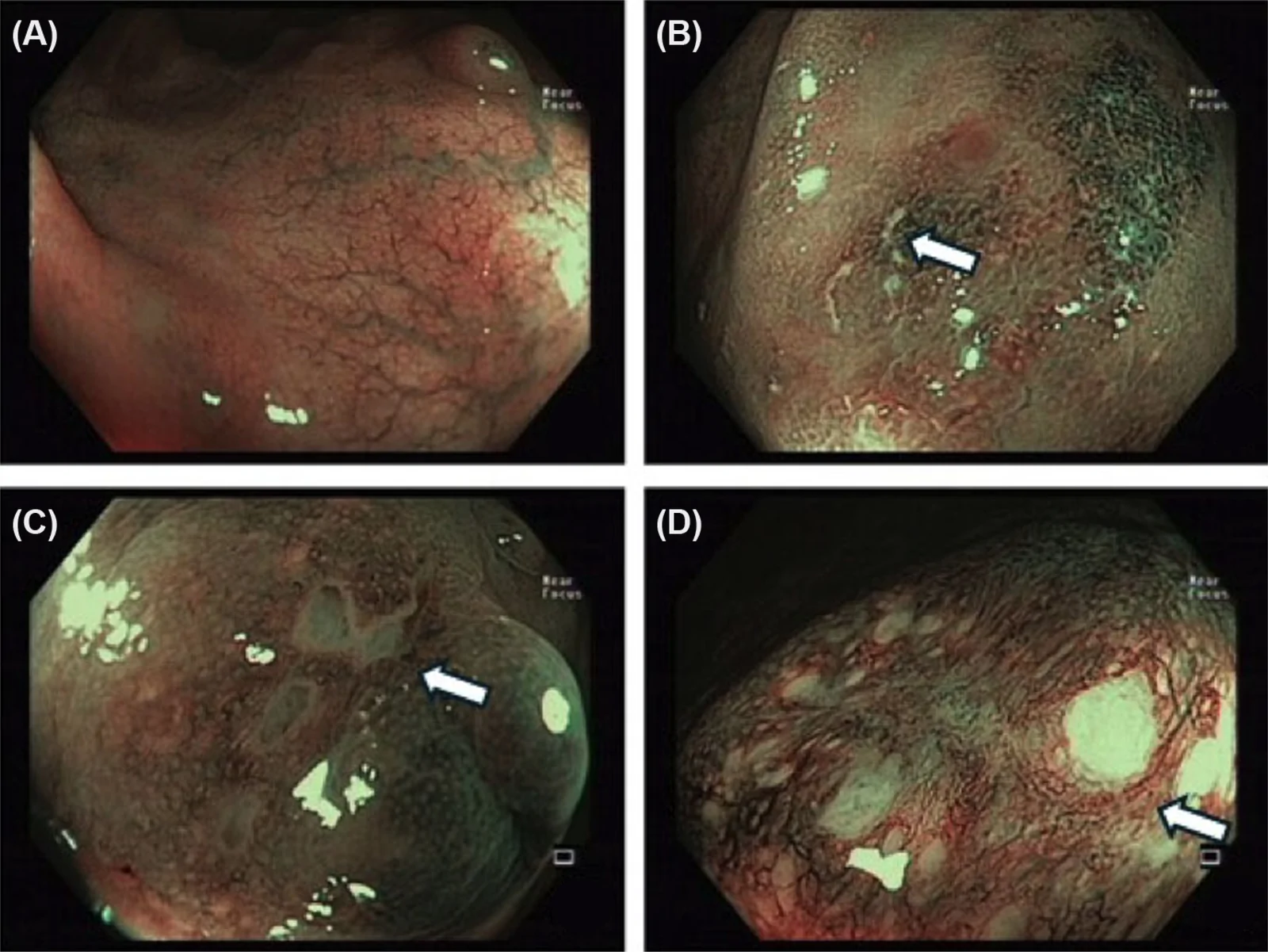
Images for each of the inflammation degrees with NBI+NF evaluation. **A**: grade 0; **B**: grade 1- microerosion; **C**: grade - erosion 3-5 mm (dots) 2; **D**: grade 3- ulcerations (dots).

**Table 1:** The characteristics of the different endoscopic scores.

-	**Grade 0 (Remission)**	**Grade 1 (Mild Activity)**	**Grade 2 (Moderate Activity)**	**Grade 3 (Severe Activity)**
**Mayo Endoscopic Subscore (WL)**	Normal mucosa and/or scars	Erythema, decreased vascular pattern, mild friability	Marked erythema; absent vascular pattern, friability, erosions	Spontaneous bleeding; ulceration
**NBI Score (NBI)**	Clearly represented vascular Pattern: brownish intramucosal vessels are clearly visible	Vascular pattern partially represented: Brownish intramucosal vessels are unclearly (partially) visible.	Vascular pattern totally not represented: brownish intramucosal Vessels are not visible; intramucosal bleeding (mucosal brownish spots)	Totally not represented vascular pattern: brownish intramucosal vessels are not visible; luminal bleeding
**NBI+NF Score (NBI+NF)**	Normal mucosa: Regular/continuous crypts, discontinuous/dilatated/elongated crypts, or crypts not visible due to scars	Microerosions <3mm, or cryptic abscesses	Erosions 3-5mm	Ulcerations >5mm

**Table 2:** Histological activity defined by nancy index.

-	**Histological Features**
**Grade 0 (Remission)**	No histologically significant disease or chronic inflammatory infiltrate (lymphocytes and/or plasma cells), with no acute inflammatory infiltrate (neutrophils).
**Grade 1 (Mild activity)**	Mild acute inflammatory cells infiltrate (neutrophils).
**Grade 2 (Moderate activity)**	Moderate or severe acute inflammatory cells infiltrate (neutrophils).
**Grade 3 (Severe activity)**	Ulcerations

**Table 3:** Demographic characteristics of the population.

Total Patients: 31	*n* (%)
Male	17 (54.8)
Female	14 (45.2)
Extensive colitis (E3)	20 (64.5)
Left-side colitis (E2)	9 (29.0)
Proctitis (E1)	2 (6.5)
Mayo Partial Score <2 (remission)	23 (74.2)
Mayo Partial Score 2-4 (mild activity)	2 (6.5)
Mayo Partial Score 5-7 (moderate activity)	6 (19.3)
Mayo Partial Score > 7 (severe activity)	0
Mesalazine	23 (74.2)
Corticosteroid	4 (12.9)
No therapy	4 (12.9)
Salazopyrine	2 (6.5)
Adalimumab	2 (6.5)
Azathioprine	2 (6.5)
Vedolizumab	1 (3.2)

**Table 4:** Agreement between endoscopic scores and histological scores.

-	Endoscopists		
	1k (95% CI)	2k (95% CI)	3k (95% CI)
**Mayo Endoscopic Subscore (WL) and Nancy Index**	0.40 (0.33-0.46)	0.35 (0.28-0.41)	0.34 (0.28-0.41)
**NBI Score (NBI) and Nancy Index**	0.78 (0.72-0.83)	0.72 (0.66-0.78)	0.81 (0.76-0.86)
**NBI+NF Score (NBI+NF) and Nancy Index**	0.42 (0.35-0.49)	0.38 (0.31-0.45)	0.48 (0.41-0.55)

**Table 5:** Inter-observer agreement.

-	Endoscopists		
	1 and 2k (95% CI)	1 and 3k (95% CI)	2 and 3k (95% CI)
**Mayo Endoscopic Subscore (WL)**	0.75 (0.68-0.82)	0.78 (0.72-0.84)	0.79 (0.73-0.85)
**NBI Score (NBI)**	0.83 (0.78-0.88)	0.91 (0.87-0.95)	0.84 (0.79-0.89)
**NBI+NF Score (NBI+NF)**	0.77 (0.70-0.83)	0.87 (0.82-0.92)	0.77 (0.71-0.84)

## Data Availability

The authors confirm that the data supporting the findings of this research are available within the article.

## LIST OF ABBREVIATIONS

CRC: Colorectal Carcinoma
HD: High Definition
MES: Mayo Endoscopic Score
NBI: Narrow Banding Imaging
NF: Near Focus
NI: Nancy Index
UC: Ulcerative Colitis
VCE: Virtual Chromoendoscopy
WL: White Light
STROBE: Strengthening the Reporting of Observational Studies in Epidemiology

## STANDARDS OF REPORTING

STROBE guidelines were followed.
